# Proof of concept for brain organoid-on-a-chip to create multiple domains in forebrain organoids†

**DOI:** 10.1039/d4ra04194a

**Published:** 2025-02-05

**Authors:** Yuan-Chen Tsai, Hajime Ozaki, Ango Morikawa, Kaori Shiraiwa, Andy Prosvey Pin, Aya Galal Salem, Kenneth Akady Phommahasay, Bret Kiyoshi Sugita, Christine Hein Vu, Saba Mamoun Hammad, Ken-ichiro Kamei, Momoko Watanabe

**Affiliations:** a Department of Anatomy and Neurobiology, University of California Irvine CA 92697 USA momokow@uci.edu; b Institute for Integrated Cell-Material Sciences (WPI-iCeMS), Kyoto University Institute for Advanced Study Yoshida-Ushinomiya-cho, Sakyo-ku Kyoto 606-8501 Japan kamei.kenichiro.7r@kyoto-u.ac.jp; c Programs of Biology and Bioengineering, Divisions of Science and Engineering, New York University Abu Dhabi Abu Dhabi UAE; d Department of Biomedical Engineering, Tandon School of Engineering, New York University Brooklyn NY 11201 USA; e Sue & Bill Gross Stem Cell Research Center, School of Medicine, University of California Irvine CA 92697 USA

## Abstract

Brain organoids are three-dimensionally reconstructed brain tissue derived from pluripotent stem cells *in vitro*. 3D tissue cultures have opened new avenues for exploring development and disease modeling. However, many physiological conditions, including signaling gradients in 3D cultures, have not yet been easily achieved. Here, we introduce brain organoid-on-a-chip platforms that generate signaling gradients that in turn enable the induction of topographic forebrain organoids. This creates a more continuous spectrum of brain regions and provides a more complete mimic of the human brain for evaluating neurodevelopment and disease in unprecedented detail.

## Abstract 2

Multilayered microfluidic chips were designed to generate morphogen gradients and topographically organized multi-region forebrain organoids.

## Graphical abstract

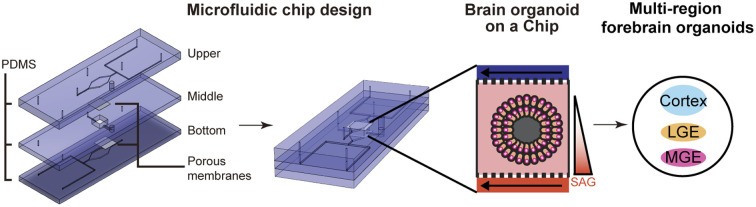

Brain organoids are self-organized three-dimensional structures derived from pluripotent stem cells (PSCs) that recapitulate many aspects of tissue structure *in vivo*^1–3^ and have tremendous potential for the study of human neurodevelopment and disease.^2,4^ However, many physiological conditions have not been achieved *in vitro*. For example, a localized group of brain cells acts as a “signaling center” during embryogenesis and creates gradients of secreted instructive signaling molecules, crucial for the formation of distinct brain regions and cell type induction.^5^ Current approaches that involve single-dose bath applications of signaling molecules are suitable for single-region organoids but lack gradients for spatially organized multi-region formation.^4,6–8^ To create multi-region structures, distinct regions must be assembled separately even though these regions normally develop together *in vivo*.^9–13^ Existing methods for generating organoids with multiple brain regions lack reproducibility, hindering controlled experiments.^2^ Thus, the field needs a more reproducible method to generate forebrain organoids that reflect the structural complexity of the human brain.

To overcome these limitations, Organ(s)-on-a-Chip (OoC) platforms (also known as microphysiological systems) provide an unprecedented opportunity to study human pathophysiology as well as developmental processes *in vitro* for disease modeling and drug discovery.^14,15^ Most OoC platforms, primarily based on microfluidic technology, provide a versatile tool for controlling environmental factors, including soluble factor gradients, 3D architecture, extracellular scaffolds, and perfusion.^16–18^ Previous studies attempted to pattern the neural tube with a morphogen gradient using microfluidic devices but mainly dealt with elongated neural tube tissue (∼15 mm width, 1.5 mm length, and 0.2 mm height) that covers the entire anteroposterior axis of the central nervous system (CNS).^19–21^ However, such designs of microfluidic devices are not suitable for producing and sustaining morphogen gradients to induce multiple domains within our smaller and spherical forebrain organoids (∼700–800 μm in diameter). Furthermore, numerous microfluidic devices have been developed to establish concentration gradients for cellular stimulation, but the perfusion flow frequently induces shear stress that damages cells.^22,23^ Therefore, there is a pressing need for devices that can apply concentration gradients to generate multiple forebrain domains in a small organoid without significant shear stresses.

We developed Brain-Organoid-on-a-Chip (BOoC) to obtain topographically organized forebrain organoids by mimicking extracellular concentration gradients. A multi-layered microfluidic device was designed to generate four gradients within an organoid culture chamber without inducing significant shear stress. We then embedded a forebrain organoid into the chamber, exposing it to a gradient of an extracellular smoothened agonist (SAG). The resulting organoid exhibited topographically organized domains, including cortical, lateral, and/or medial ganglionic eminence-like regions in one organoid. In contrast, bath application of SAG to forebrain organoids resulted in stochastic domain formation or a single domain under high concentration. Together, we demonstrate proof-of-concept that topographically organized forebrain organoids can be created using BOoC technology.

## Results

### Device design and fabrication

**Fig. 1:**
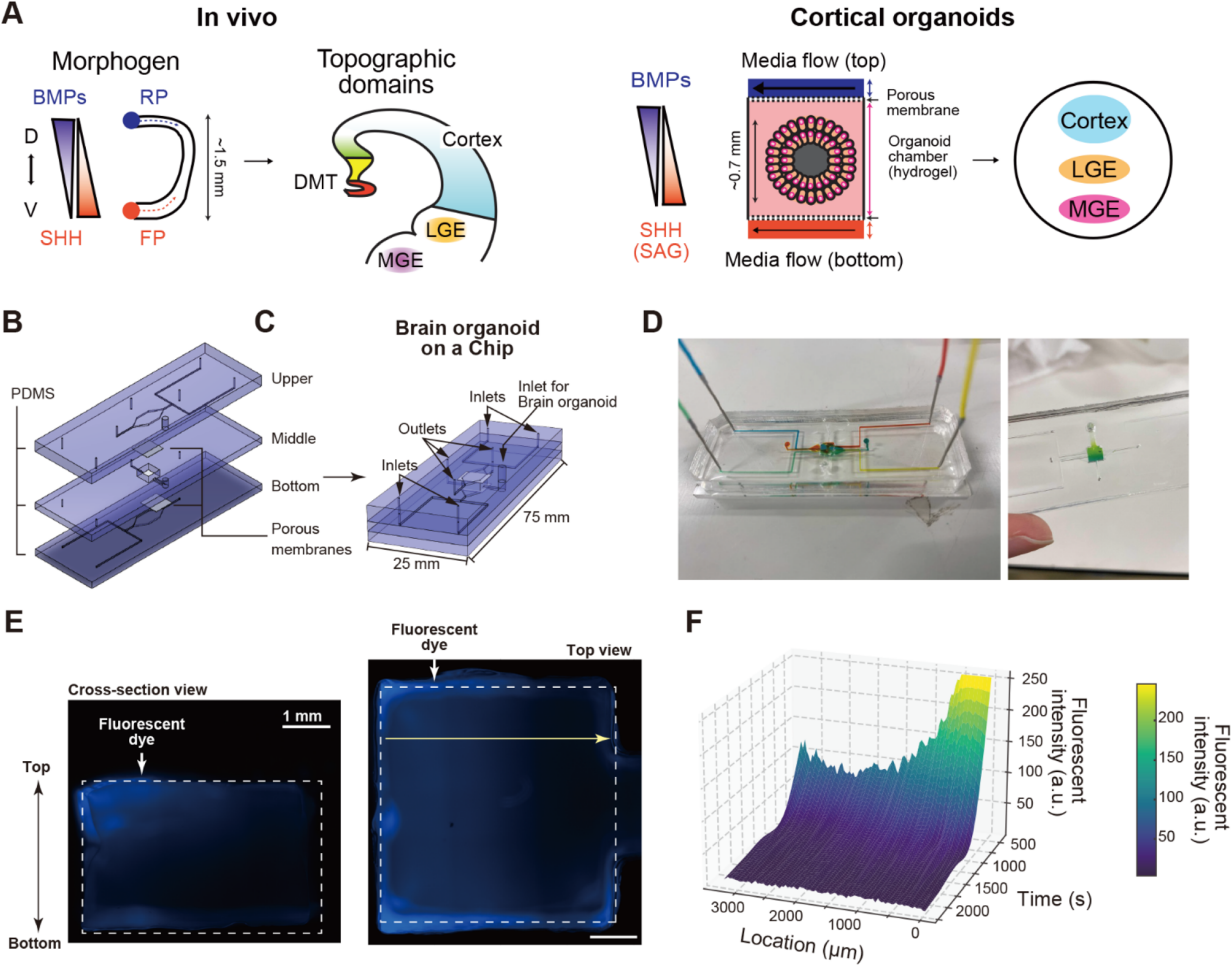
Brain organoid on a Chip. (A) Conceptual illustration of brain-organoid-on-a-chip (BOoC) to form a forebrain organoid with multiple domains. During forebrain development, signaling molecules (morphogen), such as BMPs and SHH, are secreted from signaling centers, the roof plate (RP) and floor plate (FR) respectively. The signaling gradients are formed to specify diverse brain regions including the dorsal midline telencephalon (DMT), cortex, lateral ganglionic eminence (LGE), and medial ganglionic eminence (MGE). (B and C) BOoC consists with 5 layers, including 3 microfluidic layers made of polydimethylsiloxane (PDMS) and 2 PET porous membranes. (D) An actual brain organoid on a chip introduced with colored food dyes to visualize microfluidic channels. (E) Fluorescent micrographs of agarose gel stained with fluorescent dye (AMCA-X) introduced by one of four inlets to visualize the concentration gradient in a gel. Scale bars represent 1 mm. (F) Quantitative fluorescent profiling of AMCA-X measured at the white arrow in E.

To generate topographically organized forebrain organoids, we designed a microfluidic device that can introduce 4 different growth factors into a single chamber where an organoid is embedded in hydrogels (Fig. 1A). The microfluidic device was placed on a glass slide and consisted of 5 layers, including 3 microfluidic layers and 2 porous membranes (Fig. 1B and C). The microfluidic layers were made of polydimethylsiloxane (PDMS), which is widely used for microdevices because of its biocompatibility, gas permeability, and transparency. The molds for the microfluidic layers were fabricated using a 3D printer (Fig. S1 and S2†).^24,25^ The middle microfluidic layer included an organoid culture chamber (5 mm (L) × 5 mm (W) × 2.5 mm (H)) to accommodate a forebrain organoid. The organoid culture chamber was sandwiched by 2 porous PET membranes (pore size 3.0 μm; pore density 1.6 × 10^6^ cm^−2^) that separated the upper and lower microfluidic channels to keep the hydrogel and an organoid within a chamber but allowed molecular diffusion from an upper or lower channel into the chamber. These porous membranes, in combination with the hydrogel, also help prevent exposure to perfusion flow, thereby reducing shear stress on the cultured brain organoid. The upper or lower microfluidic layers had 2 inlets to introduce 2 kinds of growth factors from each layer, leading to up to 4 growth factor inputs.

To maintain the location of the forebrain organoid in the chamber, thermoresponsive hydrogels [HG, a copolymer of poly(*N*-isopropyl acrylamide), and poly(ethylene glycol) (PNIPAAm-PEG)]^26–28^ were used. Since the sol–gel transition of these hydrogels can be regulated *via* a temperature change, the organoid can be seeded in the chamber or harvested from the chamber at a low temperature (<20 °C) and cultured in the gelated hydrogel at 37 °C. Culture medium with or without signaling molecules was diffused through the upper and lower channels through the porous membranes without significant shear stress to an organoid. In addition, the hydrogel provided sustainable 3D scaffolds that made it easier to generate and maintain signaling molecule concentration gradients.^25^

To confirm concentration gradients in the organoid chamber, food dyes, [6-((7-amino-4-methylcoumarin-3-acetyl)amino)hexanoic acid] (AMCA-X) and rhodamine 6B (Rho6B) fluorescent dyes were added to the medium in the device (Fig. 1D, E, S3 and S4,† respectively). The food dyes allowed visualization of perfusion flow in the chip. The dyes traversed through their individual channels and separately entered the organoid chamber *via* each corresponding outlet. To visualize how soluble factors diffuse across the hydrogel in the organoid chamber, a solution with AMCA-X dye was introduced into one of the inlets while the other three inlets lacked dye but were at the same flow rate (1.0 μL min^−1^). The AMCA-X dye penetrated through the PET porous membrane and reached the hydrogel in the organoid chamber, demonstrating concentration gradients within the chamber (Fig. 1F). Additionally, an increase in AMCA-X fluorescent intensity was observed after loading. Moreover, instead of using SAG, we also employed Rho6B fluorescent dye, which has a molecular weight (479.2 g mol^−1^) similar to that of SAG (490.1 g mol^−1^). Long-term monitoring showed that the diffusion and concentration gradient of Rho6B remained observable even after 4 days (Fig. S4†). Thus, the soluble factors were able to access the organoid chamber.

### Topographic forebrain organoids

**Fig. 2:**
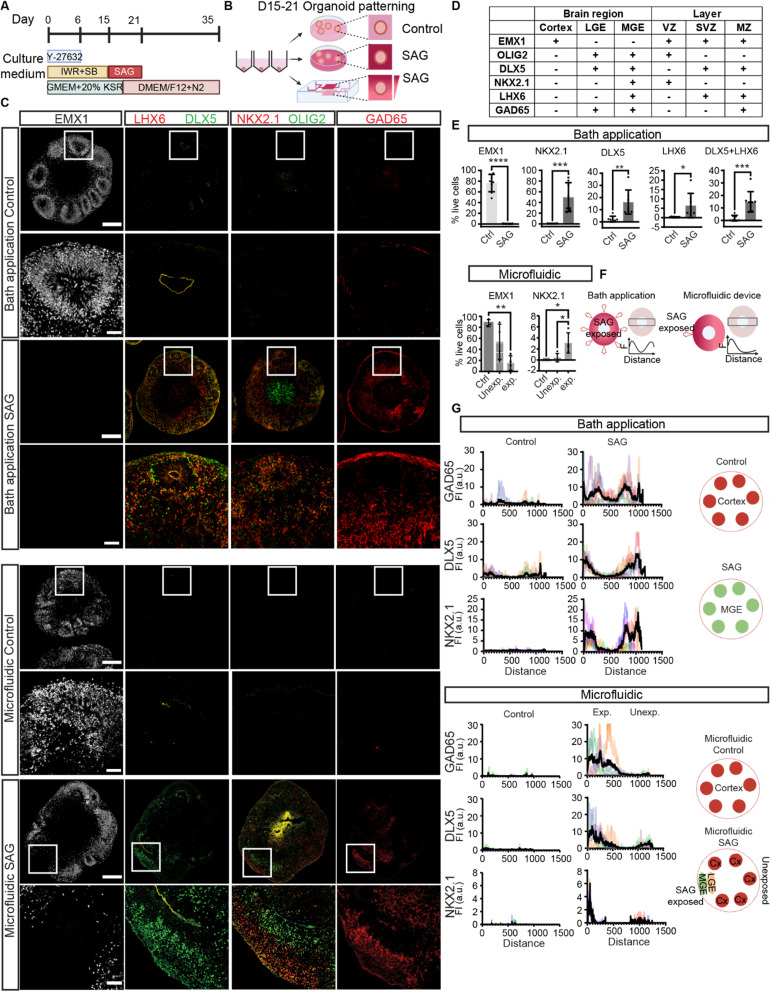
Generating topographic organization in the forebrain organoids. (A) The illustration of culture conditions for the cerebral brain organoids from day 0–35. (B) The schematic diagram of the experimental design. The SAG exposure, either with bath application or using microfluidic chips, was carried out from 15–21 days in culture. (C) Immunohistochemical analyses of forebrain organoids from bath control, bath SAG, microfluidic control and microfluidic SAG groups. Organoids from control and experimental groups were sectioned and stained for region- and cell-type-specific markers. Lower panels: zoom-in images of the box regions. Cortex marker: EMX1; GE markers: DLX5 and OLIG2; MGE markers: LHX6, NKX2.1; Inhibitory neuron marker: GAD65. Scale bars: 250 μm (whole), 50 μm (zoom-in). (D) List of cell-type- and region-specific markers used in this study and their expression patterns. (E) Percentage of live cells with region-specific markers in organoids from bath application control (*n* = 8), bath SAG (*n* = 8), and microfluidic control (*n* = 3) and microfluidic SAG (*n* = 4). Ctrl: control, Unexp: unexposed side, exp: exposed side. Bath application statistics: unpaired *t*-Test. Microfluidic chip statistics: one-way ANOVA. *p ≤ 0.05, **p ≤ 0.01. (F) Illustrations of topographic quantifications of organoids from bath application groups and microfluidic chips. Fluorescent intensity is measured across the organoids from edge to edge. Height of the box: 250 μm. (G) Topographic quantifications of cell-type- and region-specific markers in organoids from bath application control (*n* = 5), SAG (*n* = 5), microfluidic control (*n* = 3), and microfluidic SAG (*n* = 4, *n* = 2 showed NKX2.1 expression). Illustrations (bottom panels) show the regional identities of the organoids. FI: fluorescent intensity, a. u.: arbitrary unit.

We previously established a highly efficient method to generate cortical organoids from human PSCs (hPSCs) (Fig. 2A and B).^4,7^ CNS induction during development requires the inhibition of Transforming Growth Factor-β (TGFβ) superfamily signaling pathways.^3^ The default identity is the most dorsal part of the brain, the cerebral cortex.^1,3^ Here, we began by inhibiting TGFβ and Wingless (WNT) pathways to direct hPSCs to a cortical identity, and the cell aggregates self-organized into early forebrain neuroepithelial cells positive for EMX1 (∼80%) at 35 days *in vitro* with some cortical lamination (Fig. 2C and D). We then activated the ventralizing signal by using Sonic Hedgehog (SHH) or small molecules acting on the SHH pathway to form additional ventral brain regions, including the lateral and medial ganglionic eminence (LGE and MGE, respectively) along the dorsoventral axis (Fig. 1A). The LGE is adjacent to the cortex and its majority becomes the striatum, important for motor and action planning, motivation, and reward perception. The MGE is further ventral adjacent to the LGE and produces the majority of GABAergic cells.^10,29^

To induce ventral forebrain, the ganglionic eminence (Fig. 1A), we first determined the concentrations of SAG needed to reliably form LGE and MGE. With bath application (directly adding the molecules to the culture medium), the SHH signaling pathway was activated for six days from D15 to D21 and characterized organoids at D35 (Fig. 2A, B and S5C†). With bath application of 250 nM SAG, the expression of a cortical progenitor marker, EMX1, was decreased (Fig. S5C†), whereas other markers such as DLX5, OLIG2, and GAD65, were upregulated, suggesting a partial LGE identity (Fig. S5C†). Application of 500 nM SAG greatly diminished EMX1-positive cells, showing a loss in dorsal identity, and markers for general GE (OLIG2, DLX5, and GAD65) and MGE (NKX2.1 and LHX6) were upregulated, indicating increased ventral characteristics (Fig. 2C and S5C†). Collectively, we found distinct concentrations of SAG that can induce cortical to LGE regions (250 nM) and MGE regions (500 nM) in an organoid with bath applications. Similarly, SHH gave a concentration-dependent upregulation of ventral markers (Fig. S5D†). Together, SHH activation enabled the induction of LGE and MGE regions in a dose-dependent manner with bath application of signaling molecules. These data suggest that a gradient of similar molecules may aid the generation of dorsal and ventral regions in a single organoid.

Next, we tested whether the application of a signaling gradient would affect the formation of dorsal and ventral domains. We loaded neuroectodermal organoids and the hydrogel solution into the organoid chamber of the microfluidic device on ice and then warmed to 37 °C for gelation (Fig. 2B). The hydrogel-embedded organoid was exposed to SAG from the upper channel from D15 to D21, creating a gradient from 500 nM SAG (Fig. 2A and B). At D21, we removed the organoid from the chamber and continued culture in a well plate in neural maintenance media until D35. We found the SAG-exposed side of the organoid had ventral character (positive for LGE and/or MGE markers and negative for cortical markers) (Fig. 2C and S6†). In contrast, the other side retained dorsal characteristics (positive for cortical markers but negative for LGE/MGE markers) (Fig. 2C and S6†).

A comparison of the organoids exposed to the SAG bath application to those exposed to the SAG gradient revealed distinct domain patterns. Organoids exposed to bath application of 250 nM SAG or 10 ng ml^−1^ SHH had both dorsal and ventral regions, but they formed stochastically in space (Fig. 2C, E, S5C and D†). Similarly, bath application of 50 ng ml^−1^ SHH induced both LGE and MGE regions stochastically (Fig. S5D†). In contrast, regions of organoids exposed to higher concentrations of SAG (500–1000 nM) in the microfluidic gradient formed ventral domains (LGE and/or MGE markers), whereas those experiencing low concentrations generated dorsal domains (cortex markers) (Fig. 2C, E and G). This precise spatial organization of ventral and dorsal domains was not consistently observed with bath application. Together, our multilayered microfluidic device enabled a gradient of SAG exposure to induce spatially organized dorsal and ventral regions.

## Discussion

The design of our multilayered microfluidic device enabled the generation of four signaling gradients into an organoid chamber with no shear stress. Furthermore, it enabled to generate and sustain concentration gradients of signaling molecules within a relatively small range for a single forebrain organoid. Using gradients in the device, we formed multi-region brain organoids by ventralizing one side of the organoid. Bath application of the signaling molecule SAG induced ventral structures, but they were not spatially organized. In contrast, exposure to a SAG gradient in the microfluidic device induced dorsal and ventral regions in a spatially organized manner. Our results demonstrate a reproducible method for generating topographically organized brain organoids with dorsoventral structures.

Developing multi-region forebrain organoids is crucial for studying human neurodevelopment and disease accurately. Different brain regions generate unique cell types for distinct functions. For example, inhibitory interneurons are generated mainly in ventral structures, MGE and caudal GE (CGE),^30^ migrating tangentially toward the cerebral cortex. Interneurons comprise about 20–30% of cortical neurons and regulate neuronal circuitry.^31^ Without inducing ventral MGE/CGE regions in cortical organoids, neural microcircuits cannot form adequately. An alternative approach is to fuse organoids of different regional identities together, creating assembloids. Fusing cortical and GE organoids increased spontaneous neuronal activity and led to more complex neuronal oscillations.^11^ However, it lacks the essential ventral-to-dorsal cell migration seen in brain development. Also, region–region communication begins at early time points, so it is desirable for brain regions to develop together in a spatially organized manner. An additional alternative approach was to focally activate the expression of signaling molecules.^32^ However, this approach still depends on the variable extent of doxycycline-induced SHH expression, and genetic tool development for different signaling pathways might require extensive efforts. Our microfluidic device can form multiple gradients of signaling molecules, raising the possibility of generating more than two brain regions simultaneously in a single organoid. This advancement is crucial for investigating neurological and psychiatric disorders, as they often involve multiple brain regions and cell types.

Key factors in the developing brain, including BMPs, FGFs, and SHH, are secreted from signaling centers, and the concentration, timing, and spatial distribution of these signaling molecules determine cell fate.^5^ For example, SHH is initially secreted from the notochord and the floor plate for ventral patterning and later from the zona limitans intrathalamica for oligodendrocyte specification.^33^ Failure to establish these centers results in holoprosencephaly, a common forebrain malformation.^34^ Our microfluidic device, with SAG supplied on one side, mimics ventral signaling, fostering spatially organized ventral structures in forebrain organoids. Whether this device effectively induces signaling center formation is yet to be investigated. Of note, the organoid chamber size is adjustable and can accommodate larger organoids that reach millimeters in diameter. At the same time, it is crucial to design it carefully to generate appropriate concentration gradients for organoids in different scales.

The hydrogel selected for this study, MeBiol, is specifically designed for culturing stem cells^35,36^ and does not induce cell toxicity in our organoids. Matrigel, another commonly used hydrogel for embedding brain organoids,^2,37,38^ caused cell migration from the organoids into the Matrigel, disrupting the cellular organization within the organoids. Additionally, we tested other synthetic hydrogels with the organoids and found an increase in cell death. Therefore, it is crucial to test various types of hydrogels to ensure that the chosen hydrogel does not compromise the viability and cellular organization of organoids.

An advantage of the device is supplying multiple signaling molecules on each side of the organoid while allowing the user control over concentration and timing. A limitation is the use of perfusion flow to generate signaling molecule concentration gradients, which consumes costly signaling molecules, restricting experiment duration. To overcome this limitation, we employed the less-expensive SAG as a substitute for SHH. However, their diffusion coefficients differ, and the use of SAG might not fully replicate the *in vivo* developmental process. Future work should seek alternative methods for concentration gradients that minimize signaling molecule consumption.

One issue with this BOoC device is its limited throughput. Since this chip allows the generation of four different concentration gradients of signaling molecules within a single organoid culture chamber, at least four inlets are required to introduce signaling molecules. Therefore, this device aims to generate a more sophisticated brain organoid with multiple domains rather than performing high-throughput experiments.

One example of organ-on-a-chip platforms is the lung-on-a-chip established by Huh and Ingber, which accommodates only one sample per chip.^39^ This chip is beneficial for recapitulating the *in vivo* physiological movements of the lung during breathing and simulating pathophysiological conditions. Despite its limited throughput, it has been successfully commercialized. Similarly, our device is advantageous for creating sophisticated *in vivo*-like environments during brain development. Indeed, brain structures need to have very intricate structures with multiple domains, which is crucial for accurately recapitulating both physiological and pathological conditions for disease modeling.

Another critical issue is the absorption of hydrophobic molecules into PDMS due to its porosity and hydrophobicity, a problem that has been extensively discussed over a long period.^40,41^ Indeed, we observed Rho6B was absorbed into PDMS. While PDMS is commonly used for prototyping microfluidic-based OoC platforms, it is less suitable for practical applications such as drug discovery and commercialization. For these applications, it is more advantageous to use alternative materials such as thermoplastics, including polystyrene and cyclo-olefin polymer.^42,43^ These polymers not only reduce the issue of molecule absorption but also facilitate mass production.

## Conclusions

In this study, we used a multilayered microfluidic device to mimic the diffusion of molecules from brain signaling centers to guide the differentiation of human organoids. With this method, we successfully generated organoids comprised of dorsal (cortical) and ventral (LGE and MGE) regions in a spatially organized manner, mimicking the brain development along the dorsoventral axis. This system is applicable to different types of organoids and can be utilized for drug delivery.

## Data availability

The data supporting this article have been included as part of the ESI.† If further information is needed, it will be provided upon request to the corresponding authors.

## Author contributions

MW and KK conceptualized the study. KK, AM and SMH designed and fabricated multilayered microfluidic devices. YCT and HO performed experiments on forebrain organoids. KS generated and maintained forebrain organoids. APP, AGS, KAP, BKS, and CHV quantified organoid datasets. YCT performed statistical analyses. MW, KK, and YCT wrote the manuscript.

## Conflicts of interest

The University of California, Irvine (MW and KK) obtained a US patent (Patent No. US12054698B2) on the brain organoid-on-a-chip devices. The rest of the authors declare no competing interests.

## Supplementary Material

RA-015-D4RA04194A-s001
